# Outcome of Surgical Management of Hemophilic Pseudotumor: Review of 10 Cases from Single‐Center

**DOI:** 10.1111/os.13174

**Published:** 2021-11-28

**Authors:** Yun‐feng Yao, Qiang Gao, Jia‐le Li, Chen‐xi Xue, Wang Fang, Jue‐hua Jing

**Affiliations:** ^1^ Department of Orthopaedic Surgery Second Affiliated Hospital of Anhui Medical University Heifei China

**Keywords:** Hemophilia, Hemophilic pseudotumors, Surgical management, Surgical resection

## Abstract

**Objective:**

To evaluate the operative methods and clinical results of surgical treatment in a case series of 10 patients with hemophilic pseudotumors.

**Methods:**

Ten patients with hemophilic pseudotumors who received surgical resection treatment in our hospital from October 2017 to June 2020 were retrospectively reviewed. All patients were hemophilia A (factor VIII deficiency).The age range was 20–51 years. Preoperative imaging examination revealed the size of irregular mass from 8.2 cm× 3.3 cm× 2.3 cm to 22.3 cm× 15.5 cm× 17.0 cm. With the supplementary of recombinant coagulation factor VIII, five cases received complete resection; one received resection and skin grafting; one received cytoreduction surgery as the pseudotumor closing to iliac vessel and nerve; three cases received complete resection and construction as bone destruction. The perioperative variables were recorded and all the patients were followed in the outpatient clinic. Clinical and radiological assessments were conducted.

**Results:**

In these patients, the average intraoperative blood loss volume was 783.1 mL (range, 240–2100 mL). Six patients received blood transfusion during perioperative period. The average duration of surgery was 140.7 min (range, 110–240 min). All wounds healed smoothly and there was no infection or chronic sinus formation. The average length of hospital stay was 16.3 days (range, 12–25 days). There is no iatrogenic vascular nerve injury in our series. Complete follow‐up was performed in all patients. Mean follow‐up duration was 14.2 months (range, 6–26 months). One patient with pseudotumor in the thigh had a recurrence 1 year after operation, then secondary operation was performed. In three cases who received complete resection and construction, patient 8 obtained bone graft and late fixation. X‐ray examination showed bone formation in the lesion at the 2‐year follow‐ups after operation. Patient 9 underwent knee replacement, his left knee showed flexion deformity in preoparation. At the last follow‐up, range of motion was improved from 0° to 40° compared with preoperative status. Patient 10 had pseudotumor in the distal femur, received long bone graft and intramedullary nail fixation.

**Conclusions:**

Surgical resection for hemophilic pseudotumors is an effective and safe method. The choice of surgical procedure must be individualized according to the localization and progress of pseudotumor.

## Graphical abstract

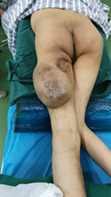

Pseudotumors occur in 1% to 2% of patients with severe hemophilia and they are associated with encapsulated blood after bleeding in soft tissue or subperiosteum. There are three kinds of pseudotumors: Type I, a simple cyst which develops within the muscle; Type II, a cyst that develops in a muscle and has an extensive blood supply in region of its attachment to a bone, which leads to compression to cortex and thinning of the cortex of bone; Type III, which starts as subperiosteal hemorrhage and destroys the structure of the underlining bone, and is considered as true pseudotumor, and originate from the bone itself. Due to the enlarged cyst resulting in compression of bone, pseudotumors often lead to bone structure erosion and fracture. Treatment for this type of hemophilic complication is very difficult due to its rarity.

## Introduction

Hemophilia is an inherited, sex‐linked, and recessive disorder of blood coagulation, characterized by prolonged bleeding and hemorrhages in the joint and soft tissues, which can be classified into types A (factor VIII deficiency) and B (factor IX deficiency). Hemophilia is prevalent worldwide and occurs in all racial and socioeconomic groups. There are more than half a million people suffering from this disease worldwide^1^. About one‐third of the cases present new mutations and no previous family medical history of hemophilia. Deficiency of clotting factors may result in spontaneous bleed or life‐threatening bleeding disorder. Its bleeding tendency and clinical symptoms are often related to the concentration of the factor and availability getting prophylaxis therapy. For hemophilic patients, especially serious cases, prophylaxis mainly concentrates in preventing of bleeding or stopping it.

Constant prophylaxis through administering the clotting factor is considered as an effective way to prevent uncontrolled bleeding and secondary tissue damage. Clinical studies have confirmed that the patients who received prophylaxis have fewer chances of spontaneous bleeding and chronic joint arthropathy and they have better joint function than those with more acute hemophilia^2^, ^3^. Giving the clotting factor is needed on a regular basis (often two to three times per week), coagulation factor replacement is a long‐term and expensive treatment option. Due to the differences in economic development, approximately 80% of patients with severe hemophilia live in developing countries, where they encounter economic constraints that prevent adequate prophylaxis^4^, ^5^. Repeated and unresolved hematomas may lead to many kinds of complications, such as pseudotumor, a serious and rare complication of hemophilia.

Hemophilic pseudotumor was first described in 1918; since then many cases have been reported. The incidence of pseudotumor is about 1% to 2% in the patients with severe hemophilia, which was found almost exclusively in men between 20 and 70 years of age. Many patients recall sustaining an injury prior to development of the pseudotumor. These lesions were usually found in soft tissues (often intramuscular) but occasionally in bone or in a subperiosteal location. It was formed as the result of repeated episodes of bleeding in bone or soft tissue^6^, ^7^. Inadequate resorption of extravasated blood results in an encapsulated area of clotted blood and necrosed tissue^8^. Pathological findings revealed most pseudotumors are encapsulated hematoma, a thick fibrous capsule surrounds a hematoma in varying states of cellular organization. Smaller cysts often have a thinner but less adherent capsule containing more fluid blood^6^. Three types of pseudotumors have been identified^9^: Type I is a simple cyst that develops within the muscle; Type II is a cyst that develops in a muscle and has an extensive blood supply in the region of its attachment to bone; Type III is considered to be a true pseudotumor and originates from the bone itself. Some authors also classify pseudotumors as proximal or distal according to their anatomical location^10^.

With successive hemorrhagic episodes, the pseudotumors expand over time and have mass effect symptoms. They cause increasing compression of adjacent structures, leading to their destruction. For example, iliopsoas hemophiliac pseudotumors may cause femoral nerve palsy, ureteric obstruction, and bowel fistulation^11^. The pseudotumors may also extend to the skin, result in fistula or ulceration. In addition to the pseudotumor of the bone itself, the bone itself may be secondarily involved. Leading factors contribute to surgical treatment of pseudotumor often involove bone structure erosion or fracture.

There were various protocols to treat hemophilic pseudotumors in the literature, including immobilization measures, replacement therapy, aspiration, curettage, radiotherapy, embolization, and surgery^12^, ^13^. Clinicians who choose replacement therapy may hope that the pseudotumor will not develop further or will even resolve, but this treatment has been proved to be ineffective for slowly growing lesions of adults. Replacement therapy combined with immobilization is beneficial to pseudotumors of small size of recent onset. Aspiration with the guidance of ultrasound is only suitable to the pseudotumor with liquid content. Curettage treatment resulted in the shrinking of the pseudotumor mass and improvement in the patient's condition, but with the risk of persistent fistula, recurrence or infection. Radiotherapy mainly apply to unresectable lesions through injury to the blood vessels feeding the pseudotumor and interference to the endothelial proliferation of the wall of the pseudotumor. In addition, radiotherapy may be a good choice for the residual pseudotumor after resection. In view of the rich vascular supply of the capsule, intra‐arterial embolization can be performed before surgery, which will reduce the amount of bleeding during and after surgery. Surgical treatment was the most effective method, especially the proximal pseudotumor^6^. However, due to the characteristics of this disease and the fewer clinical cases, treatment of complex hemophilic pseudotumor is still a challenge for orthopaedists.

The aims of this study were to investigate: (i) the outcome of surgical management of hemophilic pseudotumor; (ii) how to determine individualized surgical plan for different types of hemophilia pseudotumor; and (iii) how to reduce the complications of pseudotumor surgery.

## Patients and Methods

### Patient Selection Inclusion and Exclusion Criteria

The inclusion criteria were: (i) adult patients with hemophilic pseudotumor with extreme impairment of local tissue; (ii) patients who underwent surgical treatment by the same team in our institution; and (iii) postoperative follow‐up for a minimum of 6 months, having complete preoperative date and follow‐up outcomes. The exclusion criteria included: (i) Patients with inhibitor formation against coagulation factor.

### Patient Characteristics

A total of 10 patients who were diagnosed with hemophilic pseudotumor underwent surgical treatment between October 2017 and June 2020. Among them, the age range was 20–51 years (average, 31.5 years). Local pain, swelling, deformity, and joint disfunction were the most common reasons for a visit to hospital. All patients were defined as hemophilia A, and seven patients had a family history of hemophilia. Three patients had history of resection treatment before admission (recurrent pseudotumor). Three patients had two or more pseudotumors on admission. All those are proximal pseudotumors, six in the thigh, two in the lower leg, two in the iliac fossa. Two patients suffered from fistula formation. No neurological impairment was found in all cases. Four patients had explicit trauma history. All characteristics of patients were recorded in Table 1, including age, typing, localization, clinical symptoms, duration of disease, and others.

**TABLE 1:** Patient characteristics, management methods, and outcomes

Patient	Age (years)	Typing	Localization	Symptoms	Duration (years)	Primary/recurrent	Management	Size (cm^3^)	Surgical time (min)	Operative blood loss (mL)	Follow‐up duration (m)	Outcome
1	39	I	Thigh	Pain, swelling	10	Recurrent	Resection	18.3 × 7.2 × 6.5	120	426	24	Resolved
2	33	I	Thigh	Pain, swelling	4	Primary	Resection	8.6 × 4.3 × 2.5	110	420	14	Recurrence and resection
3	25	II	Pelvis	Pain, swelling	5	Primary	Resection	15.8 × 12.3 × 8.5	130	625	10	Resolved
4	20	I	Lower leg	Swelling	3	Primary	Resection	8.2 × 3.3 × 2.3	110	320	11	Resolved
5	22	I	Thigh	Pain, swelling	3	Primary	Resection	17.5 × 4.7 × 6.7	135	510	6	Resolved
6	51	I	Thigh	Ulceration, fistules	6	Primary	Resection + skin grafting	12.0 × 11.9 × 9.8	126	720	9	Resolved
7	43	II	Pelvis	Pain, swelling	8	Recurrent	Resection	21.0 × 11.9 × 6.8	128	920	12	Resolved
8	32	II	Lower leg	Pain, swelling	5	Primary	Resection + construction	8.6 × 3.4 × 4.0	116	240	26	Resolved
9	23	III	Distal femur	Pain, fistules, disfunction	3	Recurrent	Resection + Arthroplasty	8.9 × 5.1 × 7.7	192	1550	24	Resolved
10	27	III	Distal femur	Pain, swelling, disfunction	4	Primary	Resection+ construction	22.3 × 15.5 × 17.0	240	2100	6	Bone graft + intramedullary nailing

Typing: According to Fernandez de Valderrama JA^5^.

### Preoperative Examination and Preparation

For the intra‐muscular pseudotumor, which can be quickly and easily assessed with ultrasound, MRI can present more definite results, including size, boundary, and the involvement of blood vessels and nerves around pseudotumor. If it appears in the ilium fossa, MRI can be used to determine whether the surrounding organs are involved. Due to the different signal characteristics of hemoglobin breakdown products, MRI appearances of pseudotumor vary depending on the timing of the bleed^14^, ^15^. The size of irregular mass ranged from 8.2 cm× 3.3 cm× 2.3 cm to 22.3 cm× 15.5 cm× 17.0 cm in these patients. For bone pseudotumors, X‐ray exam can find bone destruction and soft tissue response generally. Extensive lytic lesion demarcated by mild calcification could be found in pseudotumor of the ilium. Osteolytic‐geographic lesion combined with irregular calcification reaction was shown in pseudotumor of the limb. Three‐dimensional computed tomography (CT) can define the outlines and extent of bone destruction. In our series, four cases showed bone destruction.

The inhibitor and concentration of coagulation factor were examined after admission. None of the patients tested positive for inhibitor against factor VIII. According to the concentration of factor VIII, seven cases were classified as severe hemophilia (<1% factor VIII:). Two were moderate hemophilia (1%–5% factor VIII). C‐reactive protein (CRP) increased slightly in two cases with but erythrocyte sedimentation rate (ESR) was normal. Two patients had negative results in bacterial culture of fistula secretions.

The dosage of recombinant coagulation factor VIII for replacement therapy was calculated in pre‐operation, which was based on the patient's weight and preoperative factor VIII concentration. The coagulation factor was given 1 h preoperatively as a single bolus infusion, and the dosage was adjusted to maintain a peak factor level of approximately 80% to 100% on the day of surgery, 80% on postoperative days 1 to 3, 60% on postoperative days 4 to 6, and 30% thereafter. Factor substitution was maintained for 14 days. The factor VIII level was monitored during surgery and postoperatively.

## Surgical Procedure

All operations were performed by one group of surgeons.

### Anesthesia and Position

The patients were placed in the supine, lateral, or prone position according to location of pseudotumor on the operating table, under general anesthesia.

### Approach and Exposure

The approach was on the middle of pseudotumor. Individualized selection of approach (including length and shape) is mainly for the removal of pseudotumor. The subcutaneous tissue and deep soft tissue were separated carefully. Part of muscle tissue was removed for intra‐muscular pseudotumors. It is important to manage and protect important blood vessels and nerves. Twenty minutes before the time of incision, tranexamic acid was carried out by intravenous injection, the dose used was 15 mg per kg.

### Performing Resection and Reconstruction

**Fig. 1:**
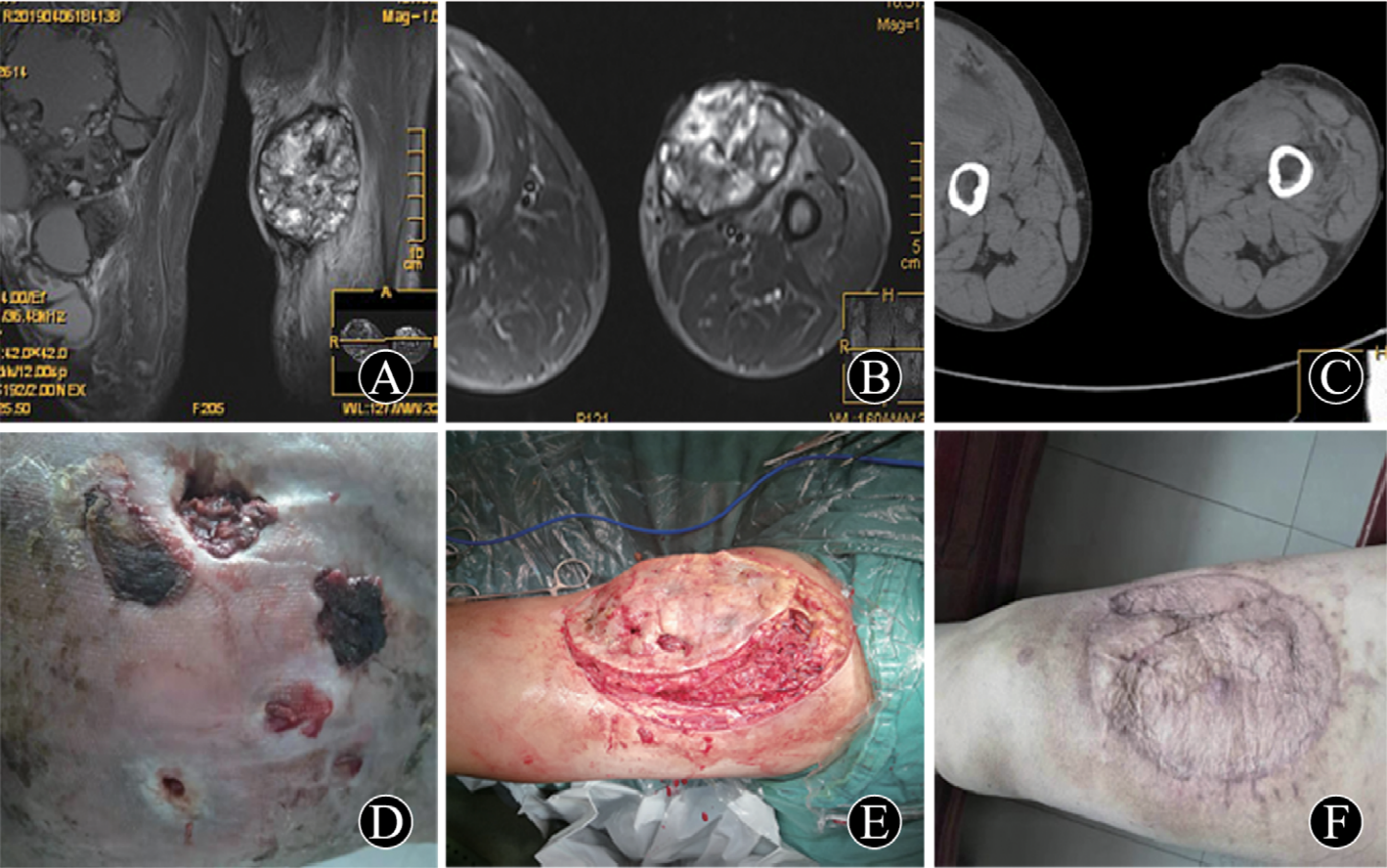
A 51‐year‐old man (patient 6), who had hemophilic pseudotumor in both thighs for 6 years, skin ulceration and fistula formation in the left thigh for 2 years. (A) Preoperative MRI T2 image in the coronal plane. (B) Preoperative MRI T2 image in the transverse plane, which showed heterogeneous signal intensity. (C) Preoperative CT image showed skin and soft tissue defect, but no bone destruction. (D) Preoperative skin ulceration and fistula formation in the left thigh. (E) Intraoperative photograph. (F) The wound healed and postoperative photograph taken at 9 months after surgery.

**Fig. 2:**
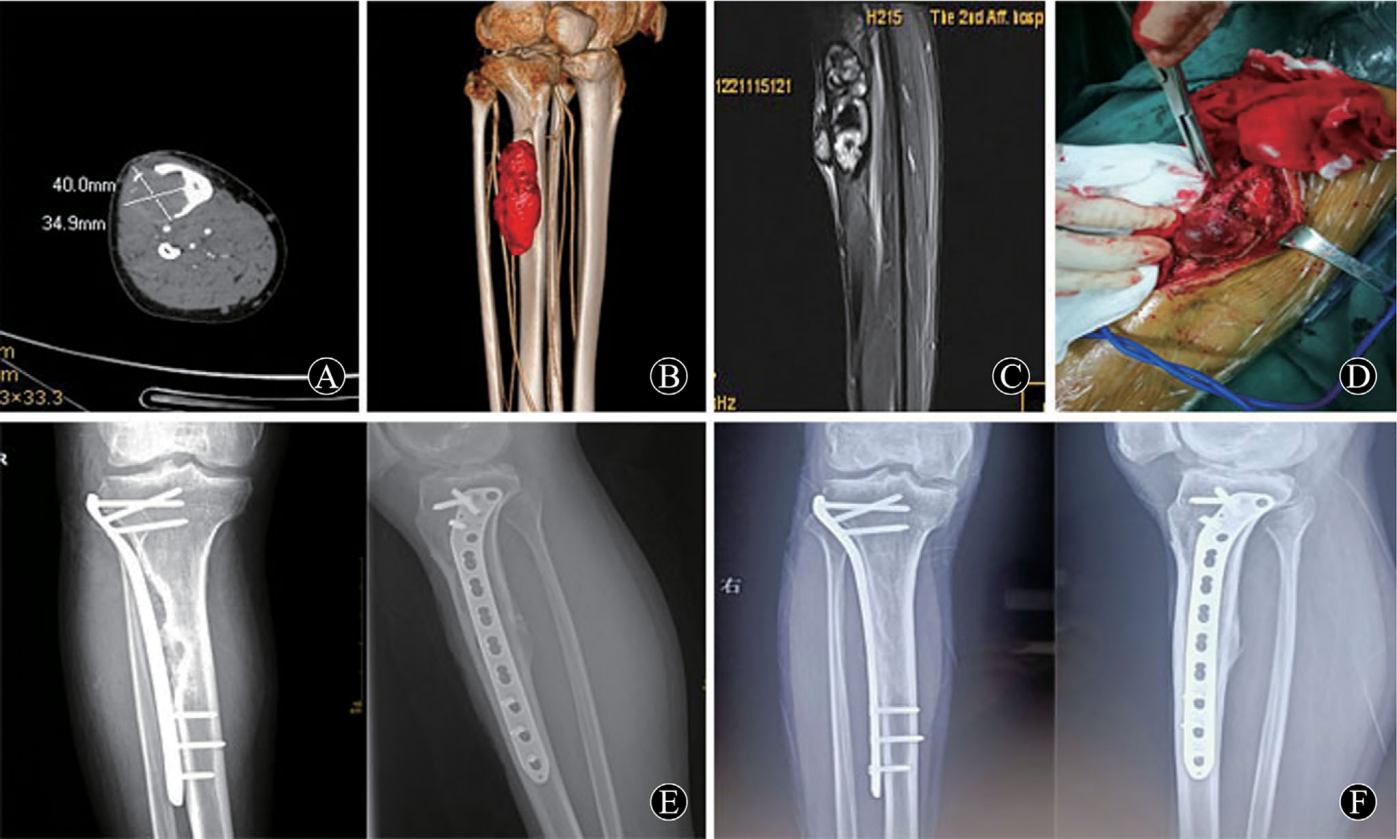
A 32‐year‐old man (patient 8) who had a 5‐year history of right lower leg progressive swelling and pain. His diagnosis was a hemophilic pseudotumor in tibia. (A) Preoperative CT image showed bone destruction. (B) Three‐dimensional CT has an important role in determining the shape and size of pseudotumor. (C) Preoperative MRI T2 image in the coronal plane, which showed heterogeneous signal intensity. (D) Intraoperative photograph showing exposure of pseudotumor. (E) Postoperative X‐ray taken at 1 month after surgery, allograft and internal fixation were performed after excision of pseudotumor. (F) Postoperative X‐ray taken at 25 months after surgery, the bone defect healed.

**Fig. 3:**
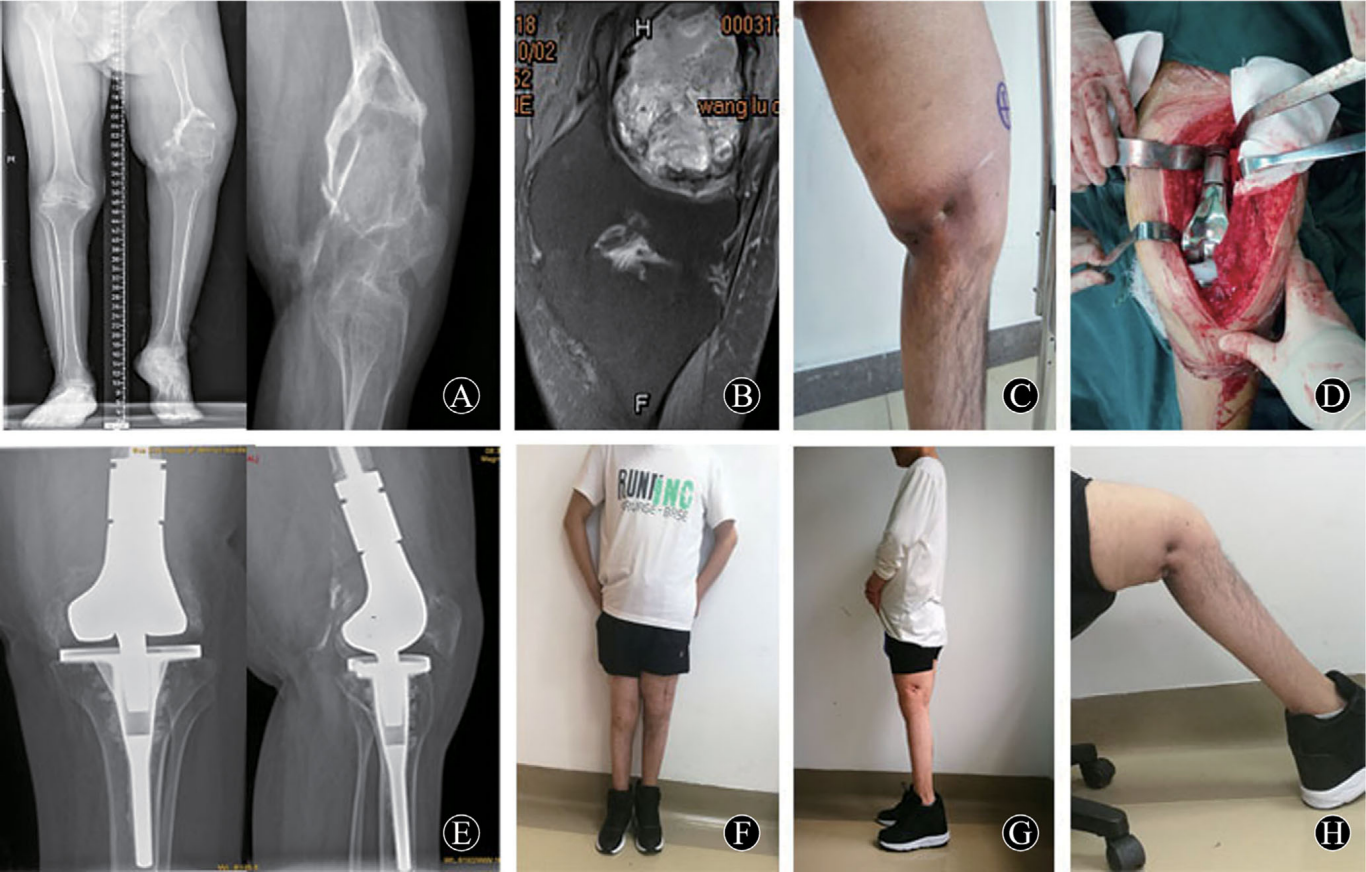
A 23‐year‐old man (patient 9) who had a 3‐year history of left distal femoral pain and progressive swelling. His diagnosis was a hemophilic pseudotumor with severe hemophilic knee arthritis. (A) Preoperative X‐ray for double lower‐limb full‐length, left lower limb was 12.0 cm shorter than the right lower limb. Preoperative lateral X‐ray showed expansile osteolytic destruction of the distal femur extending to the articular surface. Multiple bone septa were present in the lesion area. (B) Preoperative MRI T2 image in the coronal plane, heterogeneous signal intensity was present. The margin of between the lesion and adjacent area tissue was clear. (C) Preoperative physical examination of left lower limb showed distal femoral swelling and two fistulas. (D) Intraoperative photograph for knee prosthesis. (E) Postoperative photograph taken 24 months after surgery, the implants were stable. (F) Photograph of standing position taken 24 months after surgery. The patient was satisfied with wearing orthopaedic shoes. (G) Photograph of lateral standing position taken 24 months after surgery, the knee fully extended to 0°. (H) The left knee could flex to 40°. The fistulas had completely disappeared.

The method of resection depends on the origin and the extent of pseudotumor involvement. Four intra‐muscular pseudotumors were removed completely including thick fibrous capsule. The deep fascia tissue should be sutured tightly as much as possible to reduce the residual cavity. Patient 3 with pseudotumor near ilium received relatively complete removal and part of ilium involved was also removed. Patient 6 had intra‐muscular pseudotumor with skin ulceration and fistula, who suffered operations three times including debridement and skin grafting (Fig. 1). For patient 7, his pseudotumor was found growing in the iliac fossa and, during had surgery 2 years ago, part of the ilium suffered from destruction. In this case, complete resection may have been difficult because the internal side of pseudotumor was close to the internal iliac vessel and nerve; therefore, partial resection was chosen. Patient 8 showed pseudotumor invaded tibia and, after complete resection, bone graft and supplementary internal fixation were performed (Fig. 2). Patient 9 was an intra‐osseous pseudotumor with a fistula in the distal femur and ipsilateral hemophilic arthritis of the knee. This patient received knee replacement surgery after resection of the pseudotumor in the distal femur. A knee mega‐endoprosthesis was implanted in a one‐stage procedure (Fig. 3). Patient 10 had pseudotumor in the distal femur, combined with serious destruction of quadriceps, femoris, and patella. A two‐stage strategy was selected: resection of pseudotumor in one stage, arthrodesis and long bone graft in the second stage.

### Postoperative Care

Six patients had drainage tubes and remained for 2 days. Postoperatively the patients were closely monitored and received prophylactic antibiotics for 2 to 5 days.

### Clinical and Radiological Assessments

All patients were followed up in the outpatient clinic. Clinical and radiological assessments were conducted. All patients were clinically examined to evaluate the presence of symptoms, including pain, swelling, and function. Recurrence of pseudotumor was defined as re‐appearance of clinical symptoms and pseudotumor formation again on MRI, or X‐ray found increased damage to bone tissue.

## Results

### Intraoperative Results

In our series, the average intraoperative blood loss volume was 783.1 mL (range, 240–2100 mL). Six patients received blood transfusion during perioperative period. The average duration of surgery was 140.7 min (range, 110–240 min).The average length of hospital stay was 16.3 days (range, 12–25 days).

### Clinical Evaluation Results

All wounds healed smoothly, no infection or chronic sinus formation. There is no iatrogenic vascular nerve injury in our series.

Complete follow‐up was performed in all patients. Mean follow‐up duration was 14.2 months (range, 6–26 months). Patient 1 had a recurrent pseudotumor in thigh, complete resection was performed in the operation; patient 2 had a pseudotumor in the thigh and had a recurrence 1 year after operation, then secondary operation was performed; patient 3 had a pseudotumor near ilium; patient 4 had a pseudotumor in thigh; patient 5 had a pseudotumor in lower leg; patient 6 obtained wound healing after operation; and these six cases had no recurrence during follow‐up. Though partial resection was chosen for patient 7, no recurrence was found for 12 months of follow‐up. All these seven patients could walk normally and engage in simple work after operation. According to the results of follow‐up, we found most of intra‐muscular pseudotumors could be completely removed and obtained satisfactory results. When the pseudotumor increases it can involve skin and bone. The involved but unimportant bone can be removed together with pseudotumor.

In three cases who received complete resection and construction, patient 8 obtained bone graft and late fixation. X‐ray examination showed bone formation in the lesion at the 2‐year follow‐up after operation. This patient achieved good limb function after operation. Patient 9 underwent knee replacement; his left knee showed flexion deformity in preoparation. At the last follow‐up, range of motion was improved from 0° to 40° compared with preoperative status. This patient can walk freely without crutches. Patient 10 had pseudotumor in the distal femur, received long bone graft and intramedullary nail fixation. Followed for 6 months after operation, the internal fixation was stable and there was no bone resorption at the bone interface. This patient can walk with the help of crutches. All results were recorded in Table 1.

## Discussion

### More and More Patients with Hemophilia Receive Reasonable Treatment in China

In China, standardized diagnosis and treatment of hemophilia lags behind developed countries. An investigation in the 1980s confirmed the prevalence of hemophilia in China was 2.73/100,000^16^. There is a lack of systematic statistics for morbidity in recent years. At present, clotting factor substitution therapy is considered as the most effective way to reduce complications of hemophilia^17^, but significantly increase medical costs. Preventive treatment was difficult to realize in the past in China; therefore, the incidence of complications may be higher in hemophilia patients in China. With the improvement of national medical insurance policy, many patients from remote and economically undevelopmented areas came to orthopaedic clinic for treatment. Our hospital is the main institution for the surgical treatment of hemophilia patients in Anhui province in China, which is why our department could receive these patients with pseudotumors in such a short period. In the past 3 years, our team performed orthopaedic operations for 52 hemophilia patients; of those, 10 patients suffered from pseudotumor and received surgical resection. Except for one case of postoperative recurrence, most patients have achieved satisfactory results.

### Individualized Surgical Plan

The patients with pseudotumor often have multiple clinical symptoms, including pain, swelling, deformity, and joint dysfunction. Cellular death and tissue necrosis can damage local normal tissues, surrounding nerves, or vessels, though mass effect may also be involved, which is similar to pseudotumors caused by contact between the surfaces of prosthetic components^18^. The treatment of pseudotumors varies and is associated with the patient's age and the type and progression of the pseudotumor. Distal pseudotumors in children may be treated primarily with long‐term replacement therapy or local radiotherapy^6^, ^19^, ^20^. Surgical removal is the primary treatment for large proximal pseudotumors or when conservative management fails^6^, ^21^. Several case reports and retrospective analyses have shown that surgical resection is an effective and reasonable treatment for pseudotumors when the patient is covered by factor replacement therapy^19^, ^22^, ^23^, ^24^, ^25^. The cases described in this group belong to proximal pseudotumors, two cases appeared to have skin and bone destruction, and the operational indication was clear.

The choice of surgical procedure must be individualized according to the localization and the progress of pseudotumor. For muscle pseudotumors, thick fibrous capsule often has obvious adhesion with the surrounding normal muscle tissue. Careful separation should be made during the operation to remove all the capsule wall. In the present cases, five patients with intra‐muscular pseudotumors underwent resection completely. For the patient with recurrent pseudotumor in the iliac fossa, which is adjacent to important blood vessels and organs, the risk of surgical separation around pseudotumor is too high. Even if the incidence of recurrence will increase, cytoreduction surgery was performed to prevent iatrogenic neurological and vascular injuries^24^. Sufficient hemostasis was performed in the operation and hemostatic gauze, gelatin sponge, or tranexamic acid could be used locally. Postoperative drainage tube was placed to prevent the formation of local hematoma.

If the bone was involved, especially long tubular bones, mechanical function may be affected after resection of pseudotumor, the reconstruction of bone defect by autogenous or allogeneic cancellous bone may be necessary. Auxiliary internal fixation is sometimes necessary to prevent fracture complications. Cortical structural strut graft can provide mechanical stability and bone stock, which is a good choice for severe bone defect. If combined with hemophilic osteoarthritis, one‐stage arthroplasty remains an optional method. As extensive bone destruction can occur, sometimes resection and reconstruction by a megaprosthesis may be an appropriate choice. There is debate in the use of cemented or uncemented megaprostheses in literature^26^. Cemented megaprostheses was used in our study because there is often significant bone loss in patients with hemophilia.

### Complications

Perioperative blood loss, pseudotumor recurrence and infection were also important matters that the surgeons considered. Though uncontrolled perioperative bleeding is a less serious problem for surgeons with therapeutic clotting factors available, the perioperative blood loss of hemophilia patients is higher than that of non‐hemophilia patients for the same kind of operation^27^, ^28^. There also has a high blood transfusion rate in our series. Standardized application of coagulation factors, precise operative procedure, and antifibrinolytic agents are all important steps to control bleeding. In view of good clinical effectiveness and safety, tranexamic acid has been widely used in orthopaedic surgery as an antifibrinolytic agent^29^. This kind of hemostatic was routinely used to reduce the bleeding in those patients. For large pseudotumors, in order to control bleeding, many reports suggested the method of arterial embolization can be used to minimize the vascularization of the pseudotumor^30^. In our series, this method was not used because of inexperience. Pseudotumor recurrence may be a tricky problem. He *et al*.^24^ retrospectively reviewed 18 pseudotumor patients who underwent surgical treatment. Over a 40‐year period in China, of those, 15 patients received resection or resection and reconstruction, and two recurrences were observed. In contrast, Panotopoulos *et al*.^31^ analyzed six patients with a hemophilic pseudotumor who were treated through surgical treatment. At the latest follow‐up after 8.4 (range, 4–24) years, no recurrence was observed. In our series, follow‐up time is shorter, with one case showing recurrence. There is no clear reason for recurrence, which may relate to incomplete resection and late bleeding. The risk of infection was higher in patients with hemophilia than in the normal population^32^, ^33^, ^34^. There is no widely accepted explanation for the high incidence of infection in patients with hemophilia. Infection may be associated with self‐injection of coagulation factor concentration with skin contamination, human immunodeficiency virus, and hepatitis C^35^, ^36^, ^37^. There are many measures to reduce the risk of infection, such as shortening the operation time, reasonably giving antibiotics, and so on. Single‐use instrumentation in the knee arthroplasty may also be a great choice for reducing infection rate due to enhanced maintenance of sterility and decreased risk of contamination^38^.

### Limitations of Study

This study has some limitations. Firstly, it is only a case series. The sample size was small and there is no comparison with other treatments. Secondly, as hemophilic pseudotumor is a rare disease, there is no definitive grade system for clinical and radiological assessments. Finally, the follow‐up period was relatively short, further follow‐up is necessary to evaluate the definitive effect, which is helpful to improve clinical experience in the treatment of hemophilic pseudotumor.

### Conclusion

In conclusion, surgical resection for hemophilic pseudotumors is an effective and safe method. The choice of surgical procedure must be individualized according to the localization and progress of pseudotumor.

## An Authorship Declaration

All authors listed meet the authorship criteria according to the latest guidelines of the International Committee of Medical Journal Editors. All authors are in agreement with the manuscript.
